# Polymer-Free Electrospinning of β-Cyclodextrin–Oligolactide for Magnolol and Honokiol Pharmaceutical Formulations

**DOI:** 10.3390/pharmaceutics17010130

**Published:** 2025-01-17

**Authors:** Diana-Andreea Blaj, Catalina A. Peptu, Mihaela Balan-Porcarasu, Cristian Peptu, Cristina Gabriela Tuchilus, Lacramioara Ochiuz

**Affiliations:** 1 “Petru Poni” Institute of Macromolecular Chemistry, 700487 Iasi, Romania; blaj.diana@icmpp.ro (D.-A.B.); mihaela.balan@icmpp.ro (M.B.-P.); 2 Faculty of Chemical Engineering and Protection of the Environment, “Gheorghe Asachi” Technical University of Iasi, 700050 Iasi, Romania; catipeptu@yahoo.co.uk; 3 Faculty of Medicine, “Grigore. T. Popa” University of Medicine and Pharmacy, 700115 Iasi, Romania; cristina.tuchilus@umfiasi.ro; 4 Faculty of Pharmacy, “Grigore. T. Popa” University of Medicine and Pharmacy, 700115 Iasi, Romania; lacramioara.ochiuz@umfiasi.ro

**Keywords:** magnolol, honokiol, cyclodextrin–oligolactide, electrospinning, nanofibers, antioxidant activity, antimicrobial activity

## Abstract

**Background:** Magnolol (MG) and honokiol (HK) are bioactive compounds extracted from *Magnolia obovata* and *Magnolia Officinalis* trees with significant pharmacological properties, including antioxidant and antibacterial activity. However, their poor water solubility and low bioavailability limit the therapeutic potential. **Methods:** To address these limitations, this study aims to develop MG and HK formulations by co-electrospinning using custom-synthesized β-cyclodextrin–oligolactide (β-CDLA) derivatives. MALDI MS and NMR were employed for the structural assessment of the β-CDLA derivatives. This polymer-free electrospinning technique utilizes the high solubility of β-CDLA to incorporate MG and HK into fibrous webs. The morphology of the resulting fibers is established by SEM and further characterized using FTIR and NMR spectroscopy to confirm the successful incorporation of MG and HK. The antioxidant activity was determined using the 2,2-diphenyl-1-picrylhydrazyl (DPPH) radical scavenging assay, while the antimicrobial activity was evaluated against several standard microorganisms (*Staphylococcus aureus*, *Escherichia coli*, *Pseudomonas aeruginosa*, and *Candida albicans*). **Results:** The MG and HK electrospun formulations were prepared using highly concentrated feed solutions in dimethylformamide (180% w/v). The resulting β-CDLA fibers, with diameters above 400 nm and an active compound content of 7% wt., exhibited enhanced long-term antioxidant activity and improved antimicrobial efficacy, including notable activity against *Escherichia coli*. **Conclusions:** This study demonstrates the potential of MG and HK-loaded β-CDLA fibrous formulations as delivery systems with prolonged antioxidant activity and notable antibacterial efficacy, providing a promising platform for biomedical applications.

## 1. Introduction

The electrospinning technique is an efficient and versatile method for obtaining nanofibers from polymer solutions (or melts) or self-assembling molecules, offering a straightforward pathway for encapsulating various molecules, including hydrophobic drugs, bioactive agents, and nanoparticles [1]. Natural materials are becoming increasingly attractive for electrospinning compared to synthetic alternatives due to their biocompatibility, biodegradability, and reduced environmental impact [2]. Biopolymers such as polysaccharides and proteins have emerged as promising candidates for yielding nanofibers with favorable properties.

In this context, oligosaccharides such as cyclodextrins (CDs) can be directly electrospun into nanofibers without the necessity for polymer addition in the electrospinning solutions, resulting in a polymer-free electrospinning process [3]. Native CDs and their derivatives have a tendency to self-organize in aqueous solutions owing to intermolecular hydrogen bonds, resulting in layered aggregates, particles, two- and three-dimensional crystalline structures, and fibers [4]. In highly concentrated solutions, a significant quantity of aggregates is formed leading to nanofibers during electrospinning [5]. The fiber formation through the electrospinning of CD molecules is attributed to the formation of interactions and aggregates between CD molecules, which somewhat resemble the entanglements between polymer chains in the feed solutions.

CDs offer a distinctive pathway for producing functionalized nanofibers with enhanced properties such as encapsulation efficiency, photo- and thermo-stability, water solubility, and improved bioavailability of the guest molecules [6]. CDs can encapsulate hydrophobic and volatile molecules [7], a property that enables the integration of bioactive compounds into electrospun nanofibers either through inclusion processes within the CD cavity or other nonspecific physical interactions. Consequently, the association between CD and different bioactive compounds resulted in fast-dissolving nanofibers suitable for diverse food, biomedical, and pharmaceutical applications [8]. Notably, Kiss et al. employed a high-speed electrospinning method to prepare doxycycline/HP-β-CD nanofiber formulations for intravenous bolus administration [9]. This approach facilitated the energy-efficient production of the solid formulation at a high productivity rate of approximately 80 g/h. Moreover, the drug concentration of the electrospun formulations was seven times higher compared with the available commercial formulations.

Various bioactive compounds have been utilized in the co-electrospinning process with CD derivatives to obtain nanofibers, with hydroxypropyl-β-CD (HP-β-CD) and hydroxypropyl-γ-CD (HP-γ-CD) among the commonly used ones [1,6,10]. These derivatives, along with methyl-β-CD and sulfobutylether-β-CD (SBE-β-CD), have demonstrated potential for integrating diverse compounds with antioxidant properties into nanofibers. Some concrete examples include resveratrol [11], vitamin A [12], curcumin [13], carvacrol [14], thymol [15], vitamin E [16], eugenol [17], vanillin [18], and geraniol [19]. Furthermore, recent studies have highlighted the capacity of custom-synthesized β-cyclodextrin–oligolactide (β-CDLA) derivatives to form nanofibers through electrospinning [20], as well as derivatives obtained from the two homologs of β-CD, namely α-CDLA and γ-CDLA conjugates [21]. Additionally, incorporating curcumin and enrofloxacin into CDLA-based nanofibers has shown the potential of the CD-oligoester derivatives [21,22]. The structural analysis and assessment of the antioxidant activity of curcumin-loaded nanofibers revealed that cavity size matters since the drug molecules were better incorporated in β- and γ-CDLA formulations [21]. Also, enrofloxacin/β-CDLA formulations were effective against *S. aureus*, *E. coli*, and *P. aeruginosa*, having a similar antibacterial activity with the pure active principle [22].

Magnolol (MG) and honokiol (HK), extracted from *Magnolia obovata* and *Magnolia officinalis* trees, have gained attention for their diverse pharmacological effects. They are bioactive compounds with low water solubility and low bioavailability, with applications in traditional Chinese medicine, food, and cosmetics [23]. These bioactive compounds exhibit various effects, ranging from anticancer and anti-inflammatory properties to anxiolytic and antidepressant effects and antioxidant activity. In addition, MG and HK have significant antibacterial activity and inhibitory effects on a wide range of microorganisms [24,25]. The antimicrobial mechanism involves damage to the cell membrane and wall, leading to higher permeability, loss of cellular components, and changes in bacterial morphology [26].

Previous research has explored the effect of encapsulating MG in both native and modified CDs, including SBE-β-CD and carboxymethyl-β-CD [27], with a primary focus on HP-β-CD [27,28,29]. The latter derivative demonstrated the highest inclusion capacity for MG, leading to significant improvements in its bioavailability [27], stability, and water solubility which increased 500 times [28], while also reducing the MG degradation by intestinal bacteria [29]. On the other hand, α-CD and carboxymethyl-β-CD failed to form inclusion complexes, and γ-CD exhibited poor encapsulation of the bioactive compound. In previous studies, MG was loaded into amino-functionalized mesoporous silica to develop a prolonged-release drug delivery system [30], and more recently, it was employed as a new monomer for synthesizing MG-based polyurethanes [31]. To facilitate wound healing, pH-responsive MG-loaded chitosan nanocapsules with sustained antibacterial and antioxidant properties have also been developed [32]. A nanofibrous composite mat comprising poly(ester-urethane) urea, silk fibroin, and MG was prepared through electrospinning, followed by post-hydrogen bond cross-linking [33]. Antibacterial assays and in vitro cytocompatibility assessments demonstrated that the incorporation of MG notably enhances the antibacterial efficacy and promotes cell adhesion and proliferation within the composite mat.

The complexation between HK and SBE-β-CD [34] or HP-β-CD [35] led to similar results. Specifically, forming an inclusion complex with HP-β-CD led to a remarkable increase in solubility compared to pure HK [35]. Moreover, the inclusion complex significantly improved the dissolution rate and oral bioavailability of HK. This bioactive compound has also been incorporated into HP-β-CD-based liposomes [36] and γ-CD-based metal–organic frameworks [37] for drug delivery. Additionally, HK was incorporated into electrospun polymeric fibrous scaffolds based on poly(lactide-co-glycolide) [38]. These scaffolds are considered ideal candidates for drug implants owing to their advantageous morphological properties, which include a high surface-to-volume ratio, flexibility, and ease of fabrication.

In this context, the current study aims to investigate the incorporation of MG and HK into nanofibers based on β-CDLA conjugates using the electrospinning process. Scanning electronic microscopy (SEM) confirms the formation of loaded β-CDLA fibers, while rheological measurements are conducted to determine the solutions’ viscosity. Structural analyses using techniques like Fourier Transform Infrared Spectroscopy (FTIR) and Nuclear Magnetic Resonance (NMR) are employed to characterize the integration of the bioactive compounds. In addition, the antioxidant properties of MG and HK in the prepared nanofibers are established using a 2,2-diphenyl-1-picrylhydrazyl (DPPH) radical scavenging assay. Also, the antimicrobial activity of the MG and HK-loaded β-CDLA nanofibers is evaluated against Gram-positive and Gram-negative bacteria and pathogenic yeast.

## 2. Materials and Methods

### 2.1. Materials

β-Cyclodextrin (β-CD; Cyclolab, Budapest, Hungary) was dried in an Abderhalden drying pistol using P_2_O_5_ under vacuum conditions at 80 °C for 72 h. Afterwards, it was stored in a desiccator over P_2_O_5_, under an Ar atmosphere. D,L-lactide (LA; Purac, Gorinchem, The Netherlands) was recrystallized from toluene, dried under a vacuum, and kept under an Ar atmosphere in a desiccator. The anhydrous dimethylformamide (DMF) and acetonitrile were supplied by Sigma Aldrich, Saint Louis, MO, USA, while diethyl ether and methanol were supplied by VWR International, Vienna, Austria. The 4-dimethylaminopyridine (DMAP) organocatalyst, also from Sigma-Aldrich, was used without additional purification. The matrix (α-cyano-4-hydroxycinnamic acid—CHCA), cationizing agent (NaI), and Amberlyst 15 hydrogen form resin needed for the MALDI MS analysis were also acquired from Sigma Aldrich, Saint Louis, MO, USA. Magnolol and honokiol (purity 98%) were purchased from New Natural Biotechnology (Shanghai, China) and utilized as received. The antioxidant activity was assessed using 2,2-diphenyl-1-picrylhydrazyl (DPPH, Sigma Aldrich, Saint Louis, MO, USA). For antimicrobial activity, standard strains from the American Type Culture Collection (ATCC) were employed, including Gram-positive *Staphylococcus aureus* (ATCC 25923), Gram-negative *Escherichia coli* (ATCC 25922), and *Pseudomonas aeruginosa* (ATCC 27853), as well as the pathogenic yeast *Candida albicans* for antifungal testing.

### 2.2. Methods

*Synthesis of β-cyclodextrin–oligolactide (β-CDLA):* The β-CDLA derivatives were prepared using a modified version of a previously published method [21]. The reaction occurred at 25 ˚C in DMF for 24 h, using 1/8 β-CD/LA and 1/1 β-CD/DMAP molar ratios. To purify the product, cold diethyl ether was used for precipitation following the removal of the organocatalyst using Amberlyst 15 resin. Subsequently, the purified β-CDLA was vacuum-dried overnight at 50 °C, yielding 88%.

β-CDLA: ^1^H NMR (400.13 MHz, DMSO-*d*_6_, δ, ppm): 5.76–5.43 (OH2, OH3), 5.14–5.13 (CH b’, OH b), 4.84 (H1, H1’), 4.51–4.20 (OH6, H6’, CH b), 3.86 (H5’), 3.64–3.51 (H2,2’,3,3’,4,4’,5,6), 1.47–1.40 (CH3 a’), 1.30–1.23 (CH3 a). MALDI MS: 2270 g/mol.

*Electrospinning:* The electrospinning process was conducted using a Spinbox instrument (Bioincia, Valencia, Spain), controlled by WinPumpTerm software, version 0.6. Nanofibrous webs were prepared using highly concentrated β-CDLA and β-CDLA/bioactive compound solutions at 180% *w*/*v*, with a 1/1 molar ratio between the components based on the molecular mass of β-CDLA determined through MALDI MS. All solutions were prepared in a 1/1 water/acetonitrile mixture by volume. Electrospinning was carried out with a flow rate of 0.25 mL/h, a needle-to-collector distance of 15 cm, and an applied voltage of 13 kV. All electrospinning experiments are presented in Table 1.

Physical mixtures of β-CDLA and bioactive compounds were prepared by brief kneading, maintaining a 1/1 molar ratio in order to be compared with nanofibrous webs.

### 2.3. Characterization

*Solution viscosity*: The dynamic viscosity of solutions was evaluated using a Physica MCR 501 rheometer (Anton Paar, Graz, Austria) equipped with a 50 mm upper plate in a parallel plate configuration. The flow curves were recorded across shear rates ranging from 0.01 to 1000 s^−1^. The viscosity data are reported at a shear rate of 100 s^−1^.

*Fiber Morphology—Scanning Electron Microscopy*: Fiber formation was analyzed using a HITACHI SU 1510 scanning electron microscope (Hitachi, Tokyo, Japan). Samples were fixed on aluminum stubs with double-sided adhesive carbon tape. For high-resolution imaging, a 7 nm gold coating was applied under vacuum using a Cressington 108 Sputter Coater. Fiber diameters were measured from the SEM images with ImageJ 1.53k software (LOCI, University of Wisconsin, Madison, WI, USA) and reported as the average ± standard deviation. Over 70 fiber segments per sample were randomly selected for analysis across three independently prepared samples to calculate the average diameter.

*FTIR spectroscopy*: Infrared spectra were acquired using a Bruker Vertex 70 FTIR spectrophotometer (Bruker, Ettlingen, Germany) configured for attenuated total reflection. Spectral data were collected at room temperature over a range of 4000–600 cm⁻^1^, with a resolution of 4 cm⁻^1^ and 64 scans averaged for each measurement.

*Nuclear Magnetic Resonance*: NMR spectra were obtained using a Bruker Avance NEO 400 MHz Spectrometer (Bruker, Rheinstetten, Germany), equipped with a 5 mm QNP direct detection probe and z-gradients. The measurements were conducted at room temperature in DMSO-d₆, utilizing standard parameter sets provided by Bruker. Chemical shifts are expressed as δ values (ppm) relative to the residual solvent peak at 2.51 ppm for ^1^H. The average oligolactide chain length was calculated using the formula:

$$1+\frac{I_{{CH}_{3}-a^{\prime }}\;}{I_{{CH}_{3}-a}},$$

where $I_{{CH}_{3}-a^{\prime }}$ and $I_{{CH}_{3}-a}$ represent the integral values corresponding to the methyl groups of the attached oligolactide (1.49–1.44 ppm for in-chain groups and 1.31–1.29 ppm for end-chain groups.

*Matrix-Assisted Laser Desorption/Ionization Mass Spectrometry*: The mass spectrum was recorded using a RapifleX MALDI TOF TOF MS instrument (Bruker, Bremen, Germany), with instrument control and data processing performed using FlexControl 4.0 and FlexAnalysis 4.0 software (Bruker). The β-CDLA sample was dissolved in 1 mL of a 1/1 (*v/v*) water/acetonitrile mixture and mixed with a Vortex-Genie 2 device (Scientific Industries, Inc, Bohemia, NY, USA). The CHCA solution was prepared in a water/acetonitrile mixture (1/1 *v/v*) at a 20 mg/mL concentration. The NaI solution was prepared at 5 mg/mL in methanol. The sample was applied to a ground steel plate using the thin-layer technique. Thus, 1 μL of CHCA solution was placed on the target and allowed to dry at ambient conditions. Then, 0.5 μL of sample solution, spiked with NaI, was applied on top of the matrix layer and allowed to dry before analysis. The mass spectrum was registered in the positive reflectron mode, with the laser power set just above the threshold to ensure consistent signal production. The “partial sample” shooting mode, covering a small area around the initial shooting point, was used to collect 18k spectra from different regions of the spot. The calibration was carried out using poly(ethylene glycol) standards. The number average molecular mass was calculated using the formula:

$$Mn=\frac{\sum_{i}^{n}I_{i}*m_{i}}{\sum_{i}^{n}I_{i}},$$

where *I_i_* represents the intensity of the monoisotopic peak corresponding to the *m/z* ratio, while *m_i_* represents the *m/z* value of the corresponding *i* peak, with *z* = 1.

*Antioxidant activity test*: The antioxidant potential of β-CDLA/MG and β-CDLA/HK fibers was assessed employing a 2,2-diphenyl-1-picrylhydrazyl (DPPH) radical scavenging assay [39]. The β-CDLA/MG and β-CDLA/HK fibers, containing an amount of 0.7 mg of the active compound according to the theoretical calculation and NMR data, were dissolved in 5 mL of ethanol. An equivalent amount of pristine β-CDLA fibers was prepared in 5 mL ethanol. The bioactive compounds’ solutions were prepared at a concentration of 3.5 mg in 25 mL ethanol. Following dissolution, all sample solutions were filtered using 0.45 μm filters and mixed with a freshly prepared DPPH solution (75 μM) in ethanol. The sample solutions were mixed with the DPPH solution at 1/11 *v/v*. The evolution of antioxidant activity was monitored over 2 h for β-CDLA/MG, β-CDLA/HK, and the bioactive compounds, respectively. The antioxidant activity of the samples was also determined after 24 h. Additionally, β-CDLA/MG and β-CDLA/HK solutions of various concentrations were kept in the dark for 24 h to determine the effective concentration 50 (EC_50_) values. The absorbance was recorded at 517 nm, corresponding to DPPH absorption. To determine the radical scavenging efficiency of β-CDLA/MG and β-CDLA/HK samples the following equation was employed:

Antioxidant activity (%) = (*A_control_* − *A_sample_*)/*A_control_* × 100,

where *A_control_* represents the starting point’s absorbance of the 2 h evolutions and *A_sample_* indicates the absorbance at subsequent time points. In the case of the DPPH test involving various concentrations of β-CDLA/MG and β-CDLA/HK, *A_control_* is the DPPH absorbance, and *A_sample_* is the absorbance of each sample.

*Antimicrobial assay*: The antimicrobial activity was assessed using the disk diffusion method [40,41]. Mueller–Hinton agar (Oxoid, Basingstoke, UK) and Mueller–Hinton agar Fungi (Biolab, Tokyo, Japan) were inoculated with suspensions of the tested microorganisms: *Staphylococcus aureus* ATCC 25923, *Escherichia coli* ATCC 25922, *Pseudomonas aeruginosa* ATCC 27853, and *Candida albicans* ATCC 10231. The turbidity of suspensions was adjusted to the 0.5 McFarland standard, corresponding to an approximate final concentration of 1 × 10^8^ CFU/mL. Sterile stainless-steel cylinders (5 mm internal diameter, 10 mm height) were placed on the agar surface in Petri dishes. All samples were tested at a 1.5 mg/mL MG/HK concentration or equivalent. Each well was filled with 100 µL of the sample solution. The plates were left for 10 min at room temperature to ensure the equal diffusion of the sample in the medium, then incubated at 35 °C for 24 h. Commercial disks containing ciprofloxacin (5 µg/disk) and fluconazole (25 µg/disk) were included for comparison, while a water/DMSO (3/1 *v*/*v*) mixture served as the negative control. After incubation, the inhibition zone diameters (expressed in mm) were measured. All tests were conducted in triplicate, and the results are presented as the average diameter ± standard deviation.

## 3. Results and Discussion

The current study is focused on nanofiber formulations with antioxidant and antimicrobial properties using β-CDLA derivatives and MG and HK. In this scope, the β-CDLA compound employed for nanofiber preparation was synthesized using a previously published method [21]. The product was analyzed using MALDI MS and ^1^H NMR to determine its molecular mass and substitution pattern. β-CDLA has high solubility in water and organic solvent mixtures compared with the native β-CD [20,21,22,42], which ensured the successful preparation of highly concentrated solutions necessary for polymer-free CD electrospinning.

**Figure 1:**
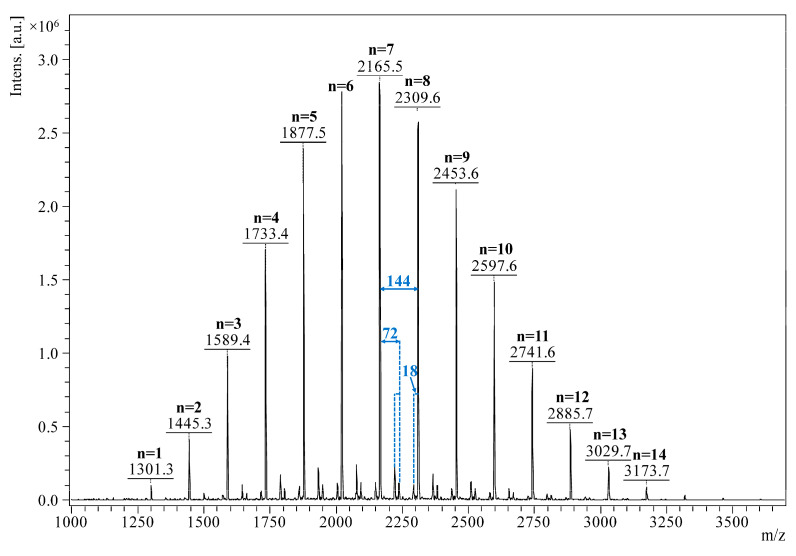
MALDI MS spectrum of the β-CDLA derivative.

The mass spectrum consists of a main peak series and several secondary series of lower intensity (Figure 1). The following equation describes the main series of peaks associated with the direct ring-opening of the lactide monomer initiated by β-CD: *m/z =* 1134 (*β-CD*) + *n* ∗ 72 (*lactate*) + 23 (Na^+^), where *n* represents an even number of lactate units attached to β-CD. One secondary series corresponds to transesterified β-CDLA species, where *n* represents an odd number of lactate units attached to β-CD. This series has a low intensity in the mass spectrum, meaning that transesterification reactions (intermolecular exchange and backbiting) were minimal in these reaction conditions. Another secondary series, down-shifted with 18 from the two mentioned series, is associated with acryl-terminated β-CDLA resulting from the elimination of water from the oligolactide chains and is described by the following equation *m/z* = 1134 (*β-CD*) + *n* ∗ 72 (*monolactate*) + 54 (*acrylate*) + 23 (Na^+^). All these series were previously observed and confirmed through MS/MS experiments [43]. The mass spectrum was further used to determine the molecular mass of 2270 g/mol, corresponding to a substitution degree of 7.73 lactide units per β-CD molecule.

**Figure 2:**
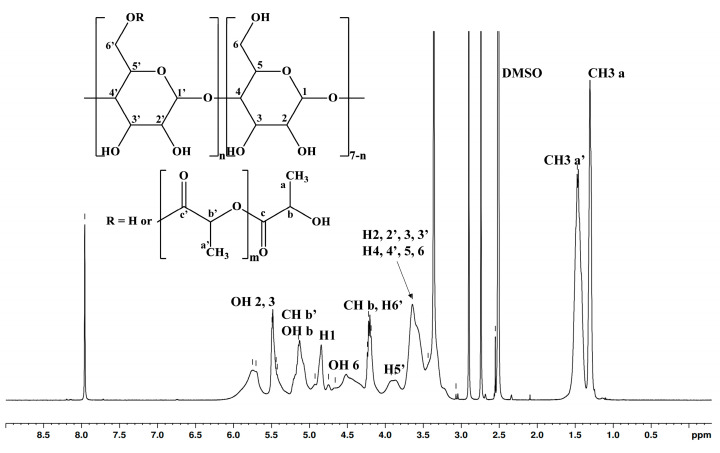
^1^H NMR spectrum of the β-CDLA derivative (DMSO-*d*_6_).

The substitution degree is challenging to determine through NMR analysis as some peaks overlap. However, the ^1^H NMR analysis (Figure 2) revealed an average chain length of 2.82 lactate units. The average number of substitution sites of 5.48 was obtained by dividing the substitution degree obtained from MALDI MS by the average chain length derived from ^1^H NMR spectroscopy. Our previous studies on β-CDLA synthesis utilizing the ^13^C DEPT 135 NMR experiment showed that β-CD is substituted at the primary hydroxyl groups [21,43,44], likely due to intramolecular exchanges within the CD molecule [45]. Figure 2 depicts the ^1^H NMR structural assessment of the synthesized β-CDLA derivative.

### 3.1. Electrospinning

The electrospinning process of native and modified CDs can be challenging as highly concentrated solutions and suitable solvents are necessary to ensure sufficient hydrogen bond interactions to obtain nanofibers from polymer-free CD solutions [3]. The electrospinning process of CDLA derivatives can be successfully performed using mixtures of water and organic solvents. In earlier studies, DMF or water/acetonitrile mixtures were employed for CDLA electrospinning [20], as well as the integration of enrofloxacin into CDLA nanofibers [22]. On the other hand, incorporating curcumin into CDLA nanofibers involved water/ethanol mixtures [21]. The concentrations used for electrospinning CDLA derivatives varied between 150% and 220% *w/v* (according to previously published studies [20,21,22]), depending on the specific derivative, the solvent mixture, and the presence of a bioactive compound or drug in the solution. For example, in the case of curcumin- or enrofloxacin-loaded β-CDLA nanofiber formulations, a higher concentration of the β-CDLA solution was required to incorporate the additional drug molecules and successfully produce the nanofibers. After testing several electrospinning conditions, highly concentrated solutions (180% *w/v*) of β-CDLA in a water/acetonitrile mixture were employed in the electrospinning process to obtain MG or HK-loaded fibers. The 1/1 molar ratio of β-CDLA to the bioactive compound corresponds to a bioactive compound mass content of approximately 7% wt.

**Figure 3:**
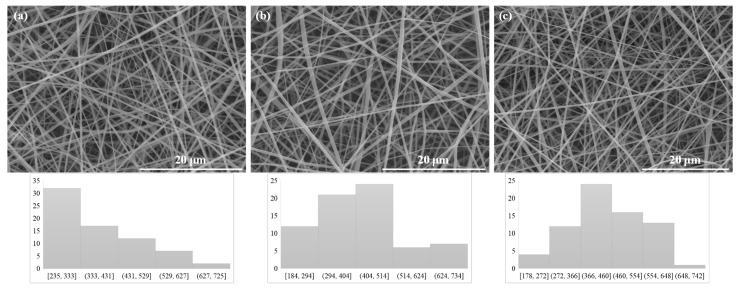
SEM images for (**a**) β-CDLA, (**b**) β-CDLA/MG, (**c**) β-CDLA/HK at a concentration of 180% *w*/*v.*

The electrospinning process of the β-CDLA/MG solution resulted in fibers with diameters measuring 416 ± 125 nm (Figure 3b), while for the β-CDLA/HK solution, fibers with diameters of 446 ± 111 nm were produced (Figure 3c). Additionally, pristine β-CDLA fibers were prepared, resulting in diameters of 374 ± 115 nm (Figure 3a). Adding a bioactive compound in the β-CDLA electrospinning solutions decreases the viscosity values, without affecting the fiber formation. Hence, the β-CDLA solution with a concentration of 180% *w/v* has a dynamic viscosity value of 1.66 Pa·s. In contrast, the MG and HK-loaded solutions had diminished viscosity values of 0.54 Pa·s and 0.557 Pa·s, respectively. Additionally, the fiber diameters obtained from the bioactive compound-loaded solutions correlate with viscosity values, with slightly higher values observed in the case of HK.

### 3.2. Structural Characterization of the Fiber Formulations

**Figure 4:**
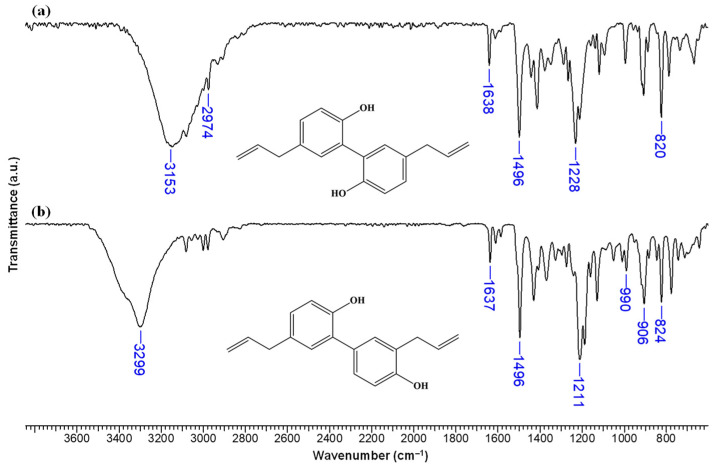
FTIR spectra of (**a**) MG and (**b**) HK.

The β-CDLA/MG and β-CDLA/HK nanofibers and physical mixtures were initially examined using FTIR spectroscopy to assess the integration of the bioactive compounds into the nanofibers compared to physical mixtures. The FTIR spectra of MG and HK closely resemble those previously reported in the literature [28,35]. The spectrum of MG exhibits strong absorption bands within the intervals 3360–2800 cm^−1^, 1640–1215 cm^−1^, and 990–780 cm^−1^ (Figure 4a). Notable absorption bands include those at 3153 cm^−1^ attributed to the O-H stretching vibration, 1638 cm^−1^ corresponding to the allyl C=C stretching vibration, and 1496 cm^−1^ associated with the C=C bond stretching vibration from the aromatic ring. Additionally, bands at 2974 cm^−1^ (C-H bond stretching vibration), 1226 cm^−1^ (C-O stretching vibration), and 820 cm^−1^ (C (aromatic)-H bending vibration) are observed.

Similar absorption bands are detected in the FTIR spectrum of HK as the two compounds are isomers (Figure 4b). At 3299 cm^−1^, the O-H stretching vibration band is noticeable, at 1637 cm^−1^, the stretching vibration of the allyl C=C bonds, and at 1496 cm^−1^, the C=C (from aromatic rings) stretching vibration band. Also, C-O bond vibrations lead to bands observed at 1211 and 906 cm^−1^, while C-C bonds appear at 990 and 824 cm^−1^, respectively.

**Figure 5:**
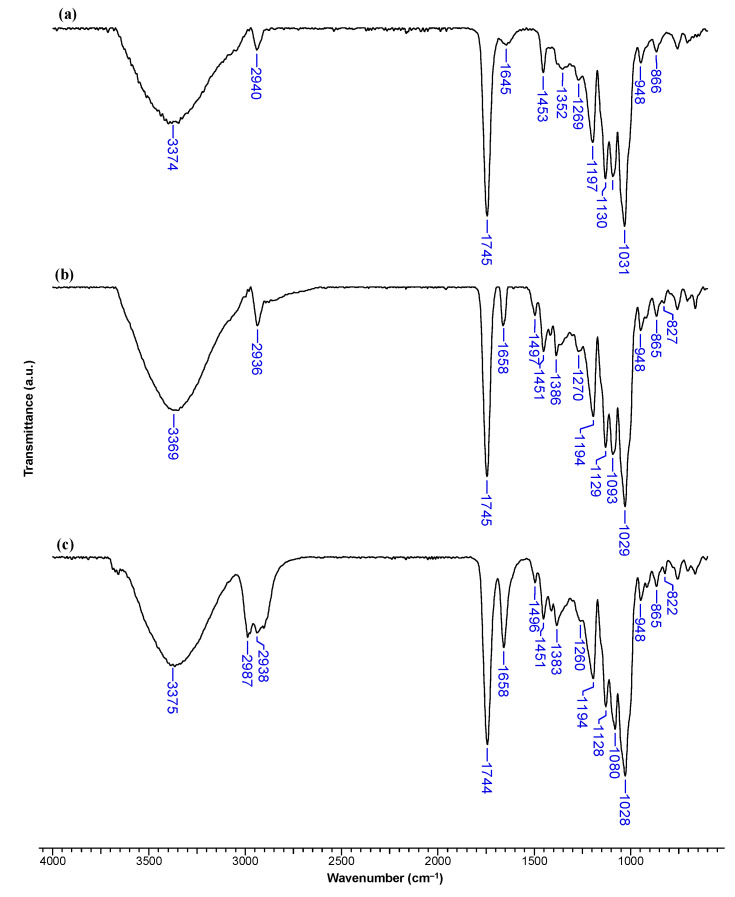
FTIR spectra of (**a**) β-CDLA nanofibers, (**b**) β-CDLA/MG nanofibers, (**c**) β-CDLA/MG physical mixture.

**Figure 6:**
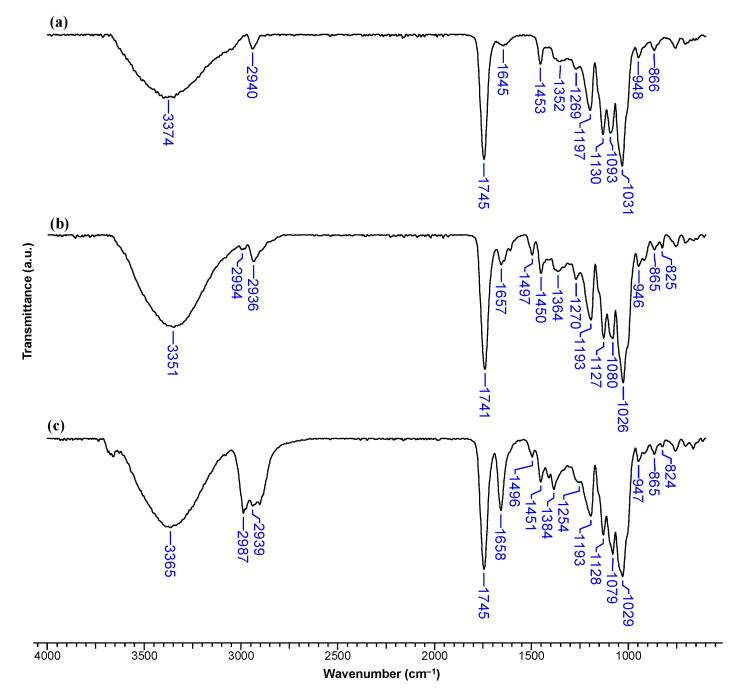
FTIR spectra of (**a**) β-CDLA nanofibers, (**b**) β-CDLA/HK nanofibers, and (**c**) β-CDLA/HK physical mixture.

The FTIR spectrum of pristine β-CDLA nanofibers resembles the one previously reported in [21] (Figure 5a and Figure 6a). In the FTIR spectrum of the β-CDLA/MG nanofiber formulations, the absorption bands specific to the β-CDLA component, found in greater quantity, are predominantly observed. However, bands associated with MG or HK can be identified at wave numbers 1497 and 827 cm^−1^ or 825 cm^−1^ (Figure 5b and Figure 6b). Similar bands associated with these vibrations are also present in the physical mixture of β-CDLA/MG and β-CDLA/HK, although at slightly lower wavenumbers than fibers, specifically at 1496 and 822 cm^−1^, respectively, at 1496 and 824 cm^−1^ (Figure 5c and Figure 6c). The absorption bands in the 1200–900 cm^−1^ region of MG and HK overlap with the most intense bands of β-CDLA. Consequently, they can no longer be observed for nanofiber formulations and physical mixtures. Some differences between nanofiber formulations and physical mixtures appear around 3000 cm^−1^ resulting from the different MG or HK distribution (homogeneous in the β-CDLA solution used for electrospinning and inhomogeneous in the physical mixture with β-CDLA powder). The absorption bands specific to the allyl group (1638 and 1637 cm^−1^ in MG and HK) overlap with the water deformation bands (around 1650 cm^−1^), thus they can no longer be observed in nanofiber formulations or physical mixtures. Cyclodextrin derivatives, usually HP-β-CD and methyl-β-CD, can interact with various drug molecules during the polymer-free electrospinning process, generally leading to the formation of inclusion complexes within the fiber matrix [1,8,10]. On the other hand, the disappearance of most bands associated with the active principles may suggest interactions between active principles and β-CDLA derivatives, probably leading to host–guest inclusion complexes, as observed in spectra of various HP-β-CD complexes [36].

**Figure 7:**
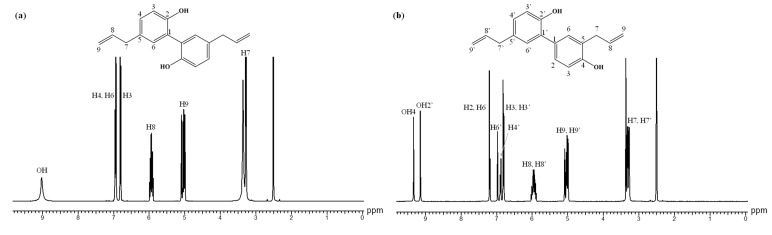
^1^H NMR spectra of (**a**) MG and (**b**) HK (DMSO-*d*_6_).

**Figure 8:**
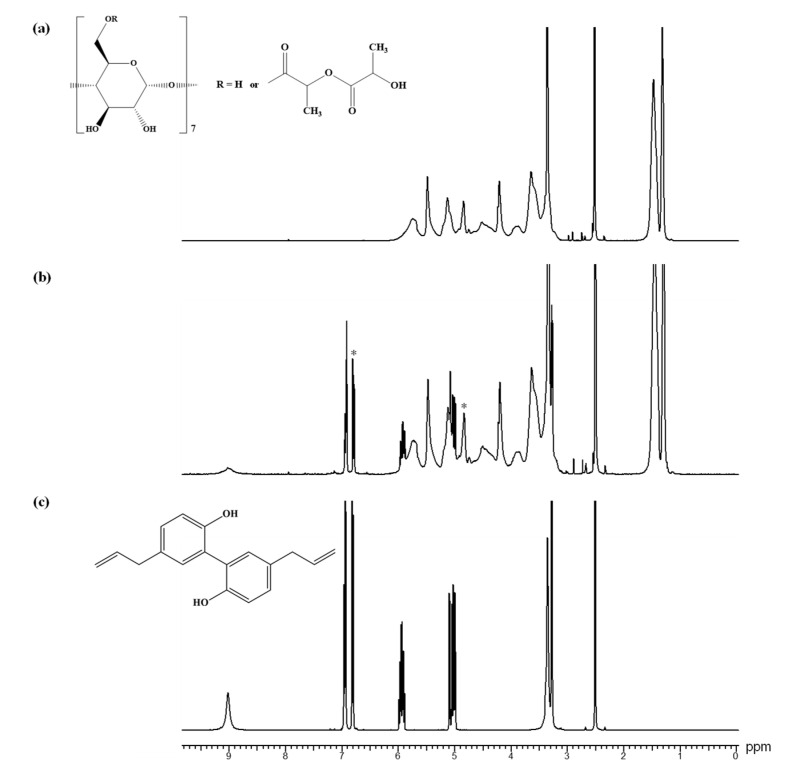
^1^H NMR spectra for (**a**) β-CDLA nanofibers, (**b**) β-CDLA/MG nanofibers, and (**c**) MG (DMSO-*d*_6_). The symbol * denotes the peaks for quantifying the β-CDLA/MG molar ratio.

**Figure 9:**
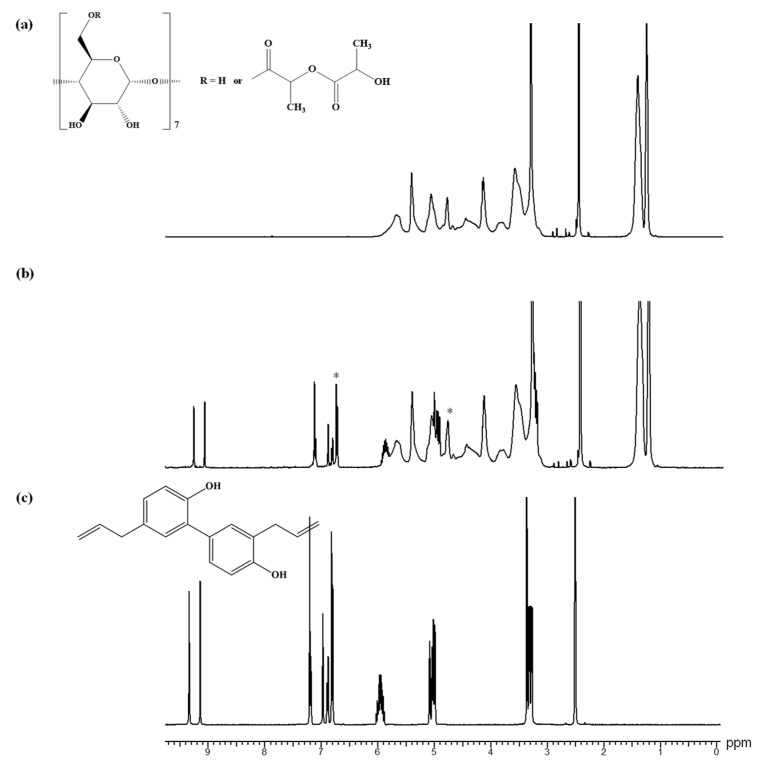
^1^H NMR spectra for (**a**) β-CDLA nanofibers, (**b**) β-CDLA/HK nanofibers, and (**c**) HK (DMSO-*d*_6_). The symbol * denotes the peaks for quantifying the β-CDLA/HK molar ratio.

Furthermore, ^1^H NMR analysis was also used to characterize the β-CDLA nanofibrous formulation of MG and HK. The peak assignments for bioactive compounds are in the ^1^H NMR spectra depicted in Figure 7. The ^1^H NMR spectrum of pristine β-CDLA nanofibers indicates that during the electrospinning process of β-CDLA solutions, the structure of the derivative is preserved (Figure 8a and Figure 9a), and no evidence of product degradation, which could lead to the formation of free oligolactide chains, is observed. The ^1^H NMR spectra of MG- or HK-loaded nanofibrous formulations present specific peaks associated with each bioactive compound (Figure 8b and Figure 9b). The bioactive compounds were added to the β-CDLA solutions in quantities corresponding to a 1/1 molar ratio. Based on NMR analysis, this molar ratio is maintained in the resulting nanofibrous formulations. The ratio between the integrals of β-CDLA anomeric protons and H3 of MG, respectively, H3 and H3’ of HK, was employed to determine the molar ratios of β-CDLA/MG and β-CDLA/HK nanofibers.

### 3.3. Antioxidant Activity

The 2,2-diphenyl-1-picrylhydrazyl (DPPH) radical scavenging test is widely employed to assess the capacity of antioxidant molecules to neutralize free radicals. DPPH is a stable free radical that gives the solution a purple color. When it interacts with molecules possessing antioxidant potential, the color of the solution changes to the characteristic yellow color of hydrazine [39]. The antioxidant activity is often linked to phenolic hydrogen groups in the molecule’s chemical structure. The reaction of these molecules with DPPH demonstrates their capability to donate a hydrogen atom to central nitrogen radicals.

**Figure 10:**
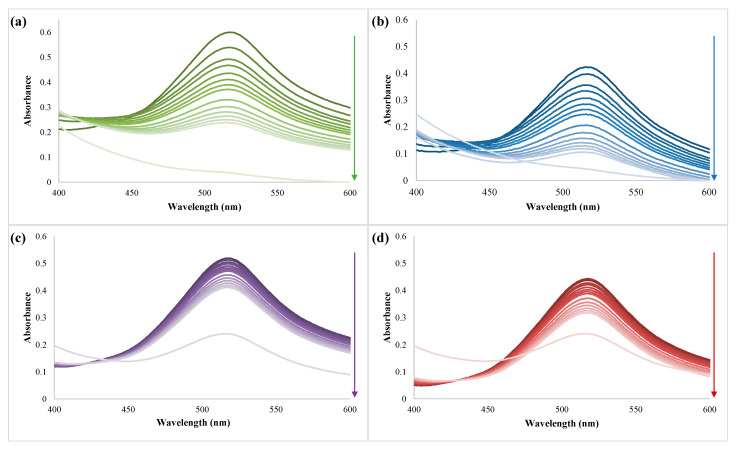
Evolution of UV-Vis spectra during the DPPH antioxidant tests of (**a**) β-CDLA/HK, (**b**) HK, (**c**) β-CDLA/MG, and (**d**) MG (the arrow indicates the direction of the absorbance evolution in time).

We aim to investigate the antioxidant activity of MG and HK-loaded β-CDLA nanofiber formulations, and the bioactive compounds themselves. All samples were dissolved in ethanol to ensure complete solubilization, and the solutions were prepared considering an amount of 7% wt. of bioactive compounds in β-CDLA formulations. Firstly, the evolution of the peak from 517 nm, which represents the absorption wavelength of DPPH, was monitored for 120 min (Figure 10). A decrease in absorbance was observed, indicating an increase in antioxidant activity, especially in the case of HK.

**Figure 11:**
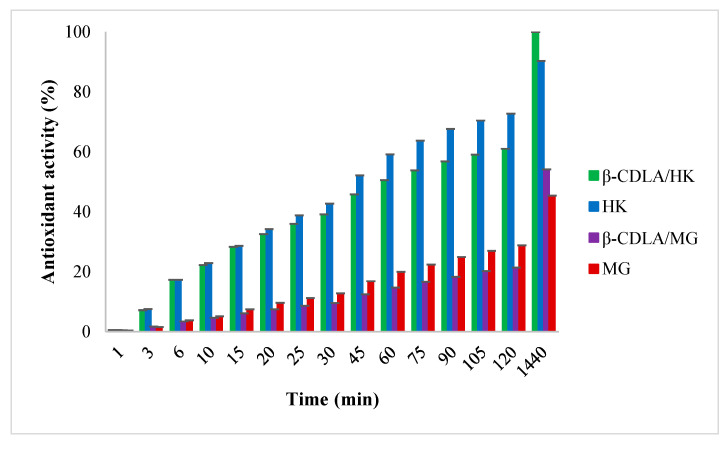
Antioxidant activity evolution of β-CDLA/HK and β-CDLA/MG nanofibrous formulations compared with the HK and MG bioactive compounds.

The ability to reduce DPPH radicals (antioxidant activity) was determined using the evolution of absorbance values in time (Figure 11). Over 120 min, HK and MG showed higher antioxidant activity values compared to the HK and MG-loaded β-CDLA nanofibrous formulations. The interaction between β-CDLA derivatives and HK or MG may prevent the hydrogen donation to DPPH radicals as a result of steric hindrance. Thus, an antioxidant activity value of 72.75% was obtained for HK and 61% for the β-CDLA/HK formulation. MG exhibited significantly lower antioxidant efficiency than HK, having a value of 29% at 120 min. Also, the β-CDLA/MG formulation led to a lower value than pure MG of only 21.4%. However, the antioxidant activity values were higher for nanofibrous formulations compared to pure HK or MG after 1440 min. Although the reduction in DPPH radicals was slower initially, the β-CDLA/HK formulation attained the maximum value of 100%, whereas HK reached 90.3%. Compared to MG, the β-CDLA/MG formulation exhibited slightly higher antioxidant efficiency, specifically 54.1% versus 45.4%. Overall, interactions between β-CDLA anf HK or MG may improve the antioxidant properties of the pure compounds in time.

In the DPPH scavenging test, the antioxidant activity value of HK surpasses that of MG, as previously observed [46]. A recent computational method study on the antioxidant activity of MG and HK as potent peroxyl radical scavengers confirmed the superior effect of HK in water media [47]. On the other hand, the reaction of MG and HK with peroxyl radicals showed that their antioxidant activity depends on the solvent [48]. This indicates that the hydroxyl groups’ hydrogens of HK are more easily available for extraction by central nitrogen radicals [46]. In the case of MG, the hydrogens from hydroxyl groups in the ortho position are able to form an intramolecular hydrogen bond, preventing the abstraction of the hydrogen by DPPH radicals. In contrast, the hydrogens from hydroxyl groups in the ortho and para positions, are not able to form such intramolecular hydrogen bonds, making hydroxyl groups of HK easily accessible for hydrogen donation to radicals.

**Figure 12:**
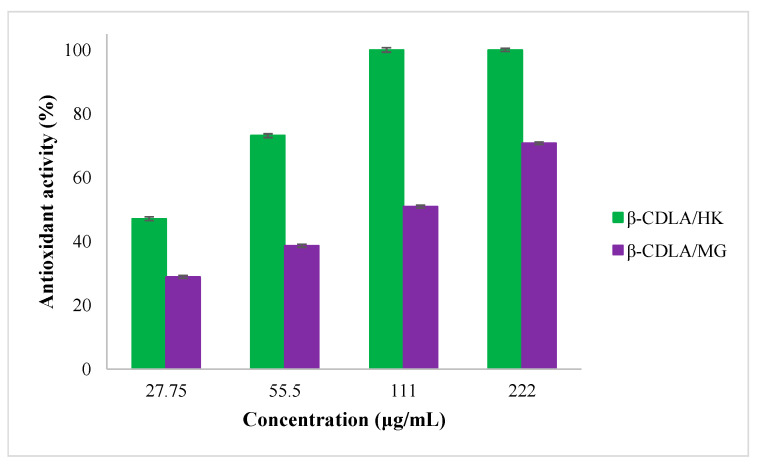
Antioxidant activity of β-CDLA/HK and β-CDLA/MG nanofibrous webs as a function of fiber concentration.

The antioxidant activity of HK- and MG-loaded β-CDLA nanofibers was also examined after 1440 min at increasing concentrations ranging from 27.75 to 222 μg/mL in ethanol to determine the efficient concentration 50 (EC_50_) (Figure 12). Thus, the EC_50_ value of β-CDLA/HK formulations is 31 μg/mL, and of β-CDLA/MG is 106 μg/mL. A lower value signifies a greater capacity for scavenging free radicals in the β-CDLA/HK nanofibrous formulations, further confirming that HK has a considerably higher antioxidant activity.

### 3.4. Antimicrobial Assay

Previous studies found that pure HK and MG were weakly active against Gram-positive bacteria and showed poor activity against Gram-negative bacteria [49]. However, when the HK and MG hydroxyl groups were conjugated with small-molecule peptide mimic fragments, all resulting derivatives exhibited significantly improved antibacterial activities against Gram-positive and Gram-negative bacteria compared to their parent compounds. The grafting of MG onto chitosan hydrochloride significantly improved the material antibacterial activity against *S. aureus* [32]. At the same time, the inhibitory effect against *E. coli* was less pronounced but still higher than chitosan hydrochloride. A recent study demonstrated that MG-loaded carboxymethyl chitosan exhibited enhanced antimicrobial activity against *S. aureus* and *E. coli* [50]. This improvement was attributed to particle formation, which increased MG’s water solubility and reduced aggregation, facilitating better contact with pathogenic bacteria.

**Figure 13:**
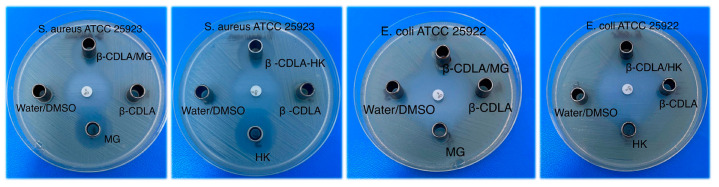
Antibacterial activity of the tested samples against *S. aureus* and *E. coli.*

The antibacterial activity of the MG or HK-loaded β-CDLA nanofiber formulations was performed using the disk-diffusion assay against standard microorganisms: Gram-positive (*Staphylococcus aureus* ATCC 25923) and Gram-negative bacteria (*Escherichia coli* ATCC 25922, *Pseudomonas aeruginosa* ATCC 27853) and a pathogenic yeast (*Candida albicans* ATCC 10231) (Figure 13). The MG and HK content was kept at a 1.5 mg/mL concentration in each sample. Table 2 shows the average inhibition zone diameter for each sample (measured in mm). Against the *S. aureus* strain, the MG and HK-loaded β-CDLA fibers and pure MG had moderate antibacterial activity, with diameters of the inhibition zone of 20 mm. Pure HK had a slightly higher effect against *S. aureus*, resulting in a 23 mm diameter. The combination with the β-CDLA derivative maintained MG antibacterial activity but slightly diminished the HK effect due to inclusion phenomena that may prevent the onset of the antibacterial activity. To ensure complete sample dissolution, a 3/1 *v/v* water DMSO mixture was utilized. Pristine β-CDLA nanofibers were used as a reference and did not present any effect against the Gram-positive bacterium. The MG and HK-loaded β-CDLA fibers were also evaluated alongside ciprofloxacin, showing its superior activity against *S. aureus*. By comparison, the antibacterial action of MG and HK-loaded β-CDLA fibers against the standard strain of *S. aureus* was inferior to that of the ciprofloxacin standard disk.

The antibacterial activity of MG and HK-loaded β-CDLA fibers against Gram-negative bacteria was tested on *E. coli* and *P. aeruginosa*, with none of the samples exhibiting an antibacterial activity against the latter strain except the ciprofloxacin standard. Pure bioactive compounds did not affect *E. coli*, a fact previously observed [26] and may be attributed to the more complex structure of the Gram-negative bacteria’s cell wall than the Gram-positive bacteria [51]. The combination with the β-CDLA derivative enabled good antibacterial activity, resulting in external diameters of the inhibition zone of 20 mm for β-CDLA/MG and 15 mm for β-CDLA/HK nanofiber formulations, respectively. Compared with the standard disk of ciprofloxacin, the MG and HK fiber formulations have significantly lower antibacterial effects.

The antifungal activity of the MG and HK-loaded β-CDLA fiber formulations was also evaluated herein against *C. albicans*. The fiber formulations had a slightly lower inhibitory effect (14.7 and 15 mm) than the pure MG and HK compounds (16 and 18 mm). Neither the water/DMSO solvent mixture nor the β-CDLA reference showed any antifungal activity. Incorporating bioactive compounds into β-CDLA fibers may reduce antimicrobial effectiveness by limiting contact with microorganisms due to strong encapsulation. Eventual degradation during processing or poor distribution within the fibers is unlikely, as the antioxidant properties are well-preserved. A lower activity is observed when comparing the effects of MG and HK formulations with the fluconazole standard disk.

Overall, the MG and HK-loaded β-CDLA fiber formulations containing a relatively high amount of bioactive compounds (7% wt.) did not significantly influence the activity against *S. aureus* and *C. albicans* compared with the pure bioactive compounds but had a positive effect against the *E. coli* strain. The inhibition zones significantly differed from those of the standard drugs but were still satisfactory. CD-based nanofiber formulations with efficient antimicrobial activity against *S. aureus* and *E. coli* were also prepared using commercially available derivatives such as HP-β-CD, HP-γ-CD, and M-β-CD and triclosan [52], geraniol [19], linalool [53], or cinnamaldehyde [54] as bioactive compounds. Concerning triclosan-loaded HP-β-CD and HP-γ-CD nanofibers, the HP-β-CD formulation had a higher inhibitory effect as the inclusion complex improved the solubility of triclosan, facilitating its efficient release into the agar medium [52]. The reduced activity of the HP-γ-CD formulation was attributed to the presence of uncomplexed triclosan, and also to the stronger interaction between HP-γ-CD and triclosan molecules which may have delayed triclosan release, further decreasing the antibacterial efficacy of the system. Therefore, we may consider that the presence of CD derivatives, although increasing the bioavailability of the drug molecules, may also induce a slight inhibition by hindering their contact with the biological target.

## 4. Conclusions

This study demonstrates the potential of β-cyclodextrin–oligolactide (β-CDLA) derivatives for developing electrospun mats loaded with magnolol (MG) and honokiol (HK), bioactive compounds known for their antioxidant and antibacterial properties. The β-CDLA derivatives were custom-synthesized and characterized through MALDI MS and NMR, which showed that β-CD was substituted at the primary hydroxyl groups with an average of 4.5 oligolactate chains, each containing approximately 2.8 lactate monomer units. These derivatives’ high-concentration water/acetonitrile solutions were then used to prepare polymer-free electrospun fibers, where hydrogen bonding interactions between β-CDLA molecules drove fiber formation. The fibers, with an average diameter of 450 nm (observed by SEM), incorporated 7% wt. of MG and HK. FTIR and NMR analysis confirmed the successful incorporation of the bioactive compounds.

Antioxidant assays showed that although the MG and HK-loaded fibers had slightly reduced antioxidant activity in the first few hours compared to the pure compounds, their performance significantly improved after 24 h, surpassing that of the native compounds. This indicates that electrospun mats provide sustained release and enhanced bioactivity over time. Furthermore, the antimicrobial activity of the fiber formulations was comparable to that of the pure bioactive compounds against *Staphylococcus aureus* and *Candida albicans*, and they also exhibited inhibitory effects against *Escherichia coli*, suggesting the advantage of the β-CDLA formulation for improving the antibacterial and antifungal properties of MG and HK.

Overall, this study highlights the potential of β-CDLA-based electrospun nanofibers as an effective delivery system for bioactive compounds such as MG and HK. Combining the β-CDLA matrix and bioactive compounds offers exciting possibilities in food chemistry, biomaterials, and pharmaceuticals, particularly for applications requiring controlled release and enhanced bioactivity. The polymer-free electrospinning process further contributes to the value of this approach by providing an eco-friendly and efficient method for fabricating these advanced materials. The findings of this study pave the way for future research into the scalability, stability, and clinical applications of bioactive-loaded nanofibers, which are potential advancements in healthcare, food preservation, and drug delivery technologies.

## Figures and Tables

**Table 1:** Electrospinning parameters for β-CDLA systems and corresponding average fiber diameters.

Sample	Viscosity (Pa·s)	Morphology	Diameter (nm)
β-CDLA/MG	0.54	fibers	416 ± 125
β-CDLA/HK	0.557	fibers	446 ± 111
β-CDLA	1.66	fibers	374 ± 115

**Table 2:** In vitro antimicrobial activity of the β-CDLA/MG and β-CDLA/HK nanofibers.

Sample	Diameter of the Inhibition Zone (mm)			
	*S. aureus* ATCC 25923	*E. coli* ATCC 25922	*P. aeruginosa* ATCC 27853	*C. albicans* ATCC 10231
β-CDLA/MG	20.0 ± 0.00	20.1 ± 0.05	0	14.7 ± 0.06
β-CDLA/HK	20.1 ± 0.05	15.0 ± 0.00	0	15.0 ± 0.00
β-CDLA	0	0	0	0
MG	20.1 ± 0.05	0	0	16.0 ± 0.00
HK	23.1 ± 0.05	0	0	18.0 ± 0.00
Water/DMSO	0	0	0	0
Ciprofloxacin	28.0 ± 0.00	33.0 ± 0.00	30.3 ± 0.57	-
Fluconazole	-	-	-	31.0 ± 0.57

## Data Availability

The raw/processed data required to reproduce these findings cannot be shared at this time as the data also forms part of an ongoing study. However, raw/processed data may be provided on request.
